# Dynamic Interactive Educational Diabetes Simulations Using the World Wide Web: An Experience of More Than 15 Years with AIDA Online

**DOI:** 10.1155/2014/692893

**Published:** 2014-01-06

**Authors:** Eldon D. Lehmann, Dennis K. DeWolf, Christopher A. Novotny, Karen Reed, Robert R. Gotwals

**Affiliations:** ^1^CMRU/NHLI, Imperial College of Science, Technology and Medicine, University of London, London SW3 6NP, UK; ^2^Interventional Radiology Unit, North West London Hospitals NHS Trust (Northwick Park & St. Mark's Hospitals), Harrow, London HA1 3UJ, UK; ^3^Department of Biological and Agricultural Engineering, North Carolina State University, NC 27695, USA; ^4^Biomedical Engineering Division, University of North Carolina at Chapel Hill, Chapel Hill, NC 27599, USA; ^5^Blue Ridge Pathology, Augusta Health, Fishersville, VA 22939, USA; ^6^Diabetes New Zealand, Rotorua, New Zealand; ^7^Shodor Education Foundation, Durham, NC 27701, USA; ^8^Department of Chemistry, North Carolina School of Science and Mathematics, Durham, NC 27705, USA

## Abstract

*Background*. AIDA is a widely available downloadable educational simulator of glucose-insulin interaction in diabetes. *Methods*. A web-based version of AIDA was developed that utilises a server-based architecture with HTML FORM commands to submit numerical data from a web-browser client to a remote web server. AIDA online, located on a remote server, passes the received data through Perl scripts which interactively produce 24 hr insulin and glucose simulations. *Results*. AIDA online allows users to modify the insulin regimen and diet of 40 different prestored “virtual diabetic patients” on the internet or create new “patients” with user-generated regimens. Multiple simulations can be run, with graphical results viewed via a standard web-browser window. To date, over 637,500 diabetes simulations have been run at AIDA online, from all over the world. *Conclusions*. AIDA online's functionality is similar to the downloadable AIDA program, but the mode of implementation and usage is different. An advantage to utilising a server-based application is the flexibility that can be offered. New modules can be added quickly to the online simulator. This has facilitated the development of refinements to AIDA online, which have instantaneously become available around the world, with no further local downloads or installations being required.

## 1. Introduction

AIDA v4 (accessible freely at http://www.2aida.org) is a downloadable program that permits the interactive simulation of plasma insulin and blood glucose (BG) profiles for teaching/demonstration/self-learning/research purposes [1]. The software incorporates a compartmental/physiological model describing glucose-insulin interaction in insulin-dependent diabetic patients (lacking endogenous insulin secretion). The graphical interface of the downloadable software allows nonspecialist users to interact with the model. AIDA v4 permits the effects of insulin dosage and dietary adjustments to be simulated for a typical patient's BG profile, with the working hypothesis being that patients, relatives, students and health-care professionals (HCPs) should be able to experience metabolic adjustments without risk of hypoglycaemia. AIDA v4 also incorporates a knowledge-based system that can suggest changes in insulin dose for users unsure about what to simulate next [2, 3].

Although a range of other interactive simulation programs of glucose-insulin interaction in diabetes have been described in the literature [4–12], to date, most of these do not seem to have been distributed so widely via the internet or been made particularly widely available. Indeed, in a number of cases, it would seem that readers are entirely dependent on the authors' own descriptions of their prototypes in research articles, since no versions appear to be available for general use by others [5, 7, 8, 12].

By contrast, with AIDA, multiple updates and versions of the software have been freely available on the web since 1996, and before that made available to researchers on diskette [13–15]. This has led to a substantial experience with the software worldwide, with over 426,000 downloads of the program taking place since the program's original internet launch.

The burgeoning popularity of the world wide web means that more people are surfing the net than ever before. This surge in popularity has led people to increasingly consult web sites as sources of *bona fide* medical information. Although there is currently a vast amount of educational material that is available on the internet, it is helpful to have *interactive* resources which engage students in the learning process. In this respect, it has been found that students learn best through active learning and discovery rather than by simply listening or reading [16, 17]. Interactive multimedia has changed the teacher/student relationship from one of “*the sage on the stage*” to the “*guide on the side*” and provides tools for students to collaborate together on projects or to work independently at their own pace and for teachers to present new types of materials [18–25].

In days gone by most computers were mainframes, and most applications were run centrally on a large host computer. Networks connected “dumb” terminals with the central mainframe. With the development of personal computers (PCs), the pendulum swung towards “distributed computing” with significant processing taking place locally on individual's desktop/notebook PCs. With the massive expansion of the internet, it has become possible once again for centralisation of functionality to be considered. Nowadays, a range of applications can operate across the internet, from distant servers. While most internet users do not have “dumb” terminals-there is a move back to using less and less complex devices for accessing the internet (e.g., mobile phones/smartphones, WebTV, etc.). It is interesting how things seem to have moved almost full circle.

With the recent move towards “cloud” computing, the interest has focused less on a single central server and more on distributed services that can be hosted on a range of servers, with more resources (bandwidth, central processing units [CPUs], memory, and disk space) commissioned as usage requires.

Using AIDA v4 as an example, work has been done to show how it is possible to move from purely static, informational resources on the web to using the internet to provide more interactive and dynamic information about clinically relevant situations in diabetes care. The purpose of the AIDA online diabetes simulator has not been to provide individual patient BG prediction or medical advice or therapy planning but instead to provide an interactive educational web-based tool to help people with diabetes and their relatives/carers, as well as HCPs and students, to better understand how meal (carbohydrate) and insulin interactions can affect BG levels; that is the intended role—for self-learning/teaching/demonstration/education purposes.

While the downloadable AIDA software has been widely applied, there may be a number of practical and theoretical limitations to the standalone AIDA PC software approach. (i) People who wish to make use of the program need to download an archive file and then install it on their hard disk. This requires a certain modicum of computer knowledge or experience. (ii) Some people might be concerned about the theoretical risk of receiving some sort of virus when downloading an executable file from the internet (notwithstanding the fact that the AIDA PC software has repeatedly been shown to be virus free). (iii) The software requires an IBM-compatible DOS/Windows PC (or an Apple Mac running SoftWindows or Virtual PC emulators or a PowerPC Macintosh). People without access to one of these computers or operating systems cannot run the standalone AIDA v4 downloadable software. (iv) The availability of enhancements or upgrades to the PC software can take time to reach end users, since further downloads and local installation are required to obtain the latest version/release. This issue could be addressed with an autoupdate facility, but such functionality is currently not yet available within AIDA v4. (v) The downloadable software, being DOS based, has a user interface that is not totally adherent to the principles of a windows graphical user interface (GUI) with which end users will be most familiar.

For all these reasons, the value of the internet has been recognised for making a diabetes simulator even more widely available than via just a downloadable program. Indeed, users might wish to try out the simulator online to see what it is all about, before going to the “trouble” of downloading and installing a standalone version.

Therefore, it was hypothesised that an even wider audience might benefit from the AIDA diabetes-simulation approach if it did not require any local download or installation, could run on a wider range of computers, and would offer a standard windows GUI for user interaction. 

To address these issues, the authors set out to collaborate on the development of a web-based version of the AIDA diabetes simulator, called AIDA online, which would be totally internet based and which therefore would be able to operate from any computer, anywhere in the world, provided it had a connection to the internet and a graphical display. The intention was that—via such a web-based simulator—enhancements and upgrades could be made available instantaneously, worldwide. Furthermore, no download or local installation would be required. In fact, it would not even be necessary to have a computer to use AIDA online, with usage being possible through any internet-enabled device, for example, WebTV or a smartphone.

## 2. Materials and Methods

### 2.1. Design Considerations

Recognising that the new target audience may be less computer “savvy” than AIDA v4 DOS users, it was planned for the input variables for the web-based simulator to be somewhat simplified with pull-down menu options used for ranges of values (e.g. “increased,” “normal” and “reduced”) instead of absolute numbers. Along similar lines, clickable terms were planned to allow unfamiliar users to see the definitions of various terms via an online glossary and also permit users to see some of the assumed values used in the simulations.

In order to make the diabetes simulator available to as many people as possible who could get on the internet, “portability” and “accessibility” were important design considerations. Given this, it was desired to make the simulator as browser friendly as possible. Web programmers distinguish “client-side” and “server-side” processing. In client-side processing, the browser asks the server to send it the program and then runs it locally. Java applets and JavaScript work like this [26].

In server-side processing, the web browser asks the server to run a program. The server does and returns the results [27]. So, with “server-side” processing, the user types data into a form, and this is sent to a web server, which calculates the results and sends them back as HTML (HyperText Markup Language) data. The developer only needs to write the program once and connect it to a web server. Then, anyone with a browser can run the program. There are no problems with porting the program to different machines [28]. Clearly, such a centralised approach might lead to issues with load on the server, although this has not been a problem to date with the web-based version of AIDA. 

Haag et al. [29] have suggested that in contrast to conventional computer-assisted instruction (CAI) programs, web-based training (WBT) programs can be considered as four main types: client-based; remote data and knowledge; distributed teaching; and server-based. Using this classification, the web-based version of AIDA would be considered a “server-based WBT program.”

In earlier days, JavaScript was not supported by many browsers. Based on this, a decision was taken to make use of “server-side” processing and translate the original AIDA v4 program source code from Pascal into Perl (Practical Extraction and Report Language) and avoid Java and Javascript.

### 2.2. Practical Extraction and Report Language (Perl)

Educational models written in Perl allow a wide variety of internet browsers to access and run simulations without requiring time-consuming downloading of lengthy files, utilising compilers, or using local disk storage space. Perl v5.0 was selected for this work for a variety of reasons, the most compelling being its portability between platforms and its widespread acceptance by web browsers. Perl is the language used in CGI (Common Gateway Interface) scripts, which are used by web servers to allow for communication with the outside world, and therefore must be recognised by web browsers. Furthermore, Perl places a minimal load on the user's system and is compatible with all major web browsers that are currently in use.

Perl is a high-level, general-purpose, nonproprietary, interpreted, and dynamic shell scripting/programming language that does not require a special linker [30]. It is a relatively simple language where variables and commands are passed to the script and output is sent directly back. In other words, Perl can be run from the command line of different platforms like DOS or UNIX and of course via the web [31].

Another reason, which was nearly as important, was the ease with which Perl could be learned. Designed to be a user friendly language and written by one person, Perl takes many of the good attributes of languages like C++, GREP, shell scripting (sh), and others to make it user friendly. Conversely, Java, for example, has been written like C and has a much steeper learning curve for first time users. This consideration factored into the decision to use Perl for this work.

Furthermore, Perl can be used without modification on multiple computer platforms. As a scripting language, it does not require compilation in order to be executed. Perl is distributed under the GNU General Public License and is maintained by a worldwide network of volunteers, a factor which also contributed to its choice for the implementation of AIDA online, which is itself a free web resource implementation of a freeware PC program.

### 2.3. AIDA Online

Designed to be accessible and usable by anyone who desires to learn more about diabetes, AIDA online has been developed to provide a windows-compatible easy-to-use web-based interface for the AIDA v4 diabetes simulator. The method of interaction between the user and the program is an important aspect of the simulator which merited consideration. The simplest method is to utilise the interaction built into HTML through the FORM function. This allows input by the user through both text and buttons, allowing a page designer to guide the user through the steps required to fill in all the necessary information to run the program. Default values can also be given to input areas to allow for the creation of a simple default example to help lead the user through what needs to be done by them to fill out the form. Using a form also lends itself to the next step, which is displaying output. Using forms is supported by the use of Perl, that can be used to create an output HTML page “on the fly,” which will incorporate both text and graphs to provide the required information for the user.

AIDA online has been designed to allow the user to run multiple simulations in order to compare the results of changes made to a patient's diet and/or insulin regimen. The design plan was for results to be viewed through the use of several different graphs that display the BG and insulin concentrations over a 24 hour period, the results of two different simulations being viewed on graphs at the same time. It was also planned for the graphs to be able to display glucose absorption, renal excretion, peripheral glucose utilization, and net hepatic glucose balance data (fluxes within the AIDA v4 model).

One of the authors, EDL, collaborated on this work with two design project students (DKDW and CAN) at North Carolina State University [25] and made the AIDA v4 Pascal source code available for translation into Perl. Under EDL's direction, the existing AIDA v4 Pascal source code was translated, and an online version of AIDA was developed in order to produce a more accessible version of the simulator for a wider (different) audience. The result of this work has become a widely utilised resource for HCPs and educators, students, patients, and relatives/carers of people with diabetes. It is freely available to anyone with internet access and has the ability to engage people from a wide variety of educational, social, and cultural backgrounds.

### 2.4. How AIDA Online Works

**Figure 1:**
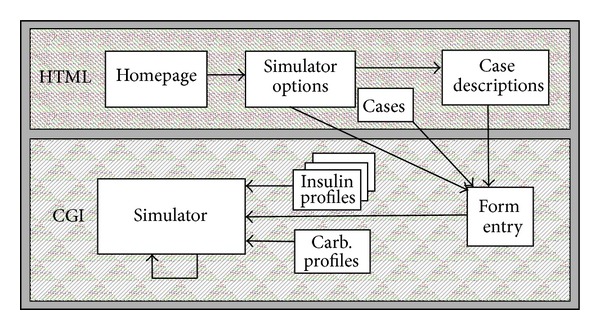
Basic structure of AIDA online. HTML = HyperText Markup Language. The AIDA online homepage (http://www.2aida.net) presents the user with various simulator options and case scenarios. A series of dedicated (Common Gateway Interface) cgi-bin scripts written in Perl v5.0 are used to read case scenario data and insulin and carbohydrate profiles from various databases. The plasma insulin and blood glucose (BG) profiles are computed using a further Perl script which contains the AIDA v4 model differential equations (and which makes use of some temporary storage space on the AIDA online server) [11]. Output from the simulator is returned to the user in HTML format for display by a web browser.

The AIDA online website utilises two divisions of a web server, the HTML public domain area and the cgi-bin script/program area (Figure 1).

The HTML area hosts all static web pages including most of the forms that post information directly as input to programs stored in the cgi-bin area of the host (AIDA online) web server. This area also hosts the graphics files and temporary numerical data files produced by the simulation engine. This allows the user, if required, to download the information produced by the simulator into local storage.

The cgi-bin Perl scripts (programs) which actually run the simulations are executed in realtime, so that they can output dynamic information (data and graphs) which can be viewed immediately through a web browser. The Perl scripts have been set up to accept input from the web server (HTML form submission) as well as from static text files stored at the website. The main output from these scripts is formatted in HTML. This allows the user's web browser to view and download the output of the AIDA online scripts. The HTML output does not actually exist physically as files but rather is created dynamically in realtime, upon demand (generated “on the fly”). The Perl scripts' other outputs include physical text files that store data used in the generated plots. There are a total of three scripts used to generate AIDA online simulations (cases.cgi, intro.cgi, and simulate.cgi). Two scripts (cases.cgi and intro.cgi) are used to process user preferences in order to set up forms for the users' input. The third script (simulate.cgi) actually computes the AIDA diabetes model and can be considered the “simulation engine”, generating the simulations.

The HTML area is also used to store all static web pages that provide information, help, and directions to the simulator.

The simulator requires several parameters that allow users to customise their simulations. For example, users can select their preferred units for BG display (mmol/L versus mg/dL), select the insulin types to be used, and decide whether or not they would like to see the advanced fluxes plots underlying the simulations (net hepatic glucose balance [32], peripheral glucose utilization, renal excretion of glucose into the urine, and glucose absorption from the gut) as part of their simulation runs.

As in AIDA v4, users can choose from 40 existing, pre-stored scenarios or create a new case from “scratch.” An abbreviated description of the cases is provided initially on the “Options” web page. However, if the user needs a further description or more details, a link is given to a web page where each of the 40 sample cases is described in full. The case details page allows the user to choose a case and return to a similar customisation page to proceed with a simulation.

**Figure 2:**
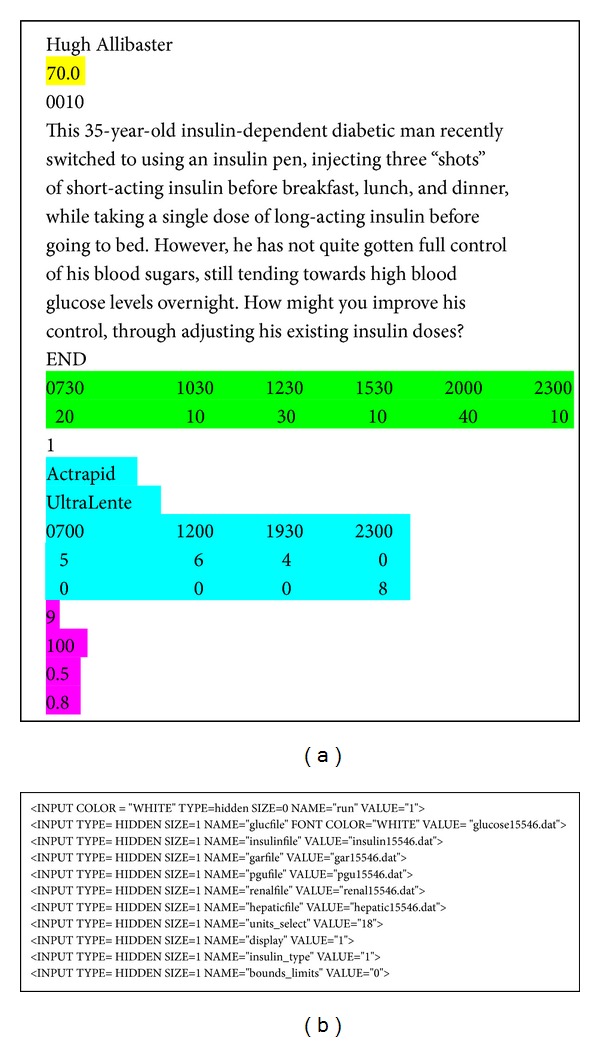
(a) Data from an ASCII (American Standard Code for Information Interchange) text file for an example case scenario (number 0010: Hugh Allibaster) from the AIDA online database. Depending which case scenario the user selects, these data are read in and used as the basis for an initial (baseline) simulation. Forty case scenarios exist in AIDA online, data imported from AIDA v4. Colour key, highlighted in figure. Yellow: weight (in kg). Green: meal times and grams of carbohydrate. Cyan: preparations, times, and dosages of insulin injections. Magenta: renal threshold of glucose, creatinine clearance rate, and hepatic and peripheral insulin sensitivity parameters, respectively. (b) Hidden fields in the dynamically generated HTML web pages produced by the AIDA online cgi-bin Perl scripts. These fields are used to store parameters and case details to be transferred between simulation runs. “units_select” value = 18 for mg/dL blood glucose units; value = 1 for mmol/L. Premixed/biphasic “insulin_type” value = 2. “display” value = 1 is standard display; “display” value = 2 is advanced/fluxes display. “run” value = 1 is the simulation run number (1 = first, baseline simulation). “run” value number increments for each subsequent simulation. “bounds_limits” value = 1 would show the user-defined normoglycaemic ranges. 5 digit number is a PID = process identification number generated by the web server for each separate process/simulation.

Once the user has selected a case scenario, a cgi-bin Perl script is invoked. This script is passed the preferences of the user and the sample case, if one has been chosen. If an existing case is chosen, the script populates an HTML template form with data extracted from static text files obtained from AIDA v4, which are stored on the AIDA online web server for that individual case scenario (Figure 2(a)). This form would be left blank if the user decides to start a new case. Using this form, clinical and nutritional data are submitted to the AIDA online simulation engine.

When the user opts to run the simulation, this HTML form data and the user's preferences are passed to the simulation engine, implemented in Perl, and which makes use of the same model as that adopted for the downloadable AIDA v4 simulator [2] to generate the BG and plasma insulin profiles.

Based on the user's time of carbohydrate intake and amount, as in AIDA v4, the simulator reads prestored values from a static text file—effectively a large lookup table—containing precomputed carbohydrate gut absorption profiles generated by one of the authors (EDL) and taken from AIDA v4. The same process occurs with the insulin injections. The time, amount, and type of insulin injections are used to determine which file and what value to extract. This data is then used by the simulator to calculate the BG and plasma insulin levels as well as each of the four fluxes at 15-minute intervals, using an Euler integration method, during a simulated 24 hour day, as per the original AIDA v4 model [2].

An early prototype version of AIDA online incorporated observed BG data points—for example, “Glucostix” measurements—as in the downloadable version of AIDA, but these were not retained when AIDA online went “live” on the web, in order to try and reduce the amount of user entered data required to run a simulation and thereby simplify user interaction with AIDA online.

Storage data files used by AIDA online were previously processed and saved in the same location as the Perl script. Eight of the data files represent the different insulin absorption profiles based on the type of insulin injected and the dosage. Four of the eight files represent the “active” insulin absorption, while the other four pertain to the plasma insulin profiles [4]. A different file represents the glucose absorption data based on the amount of carbohydrates ingested. The data arrays of absorption profiles stored in these files are the product of a precomputation program written by Lehmann and Deutsch [2]. The storage file was originally written in binary for the DOS version of AIDA which allowed for fast access to the data. However, for AIDA online, a decision was taken to have the storage files written in an ASCII (American Standard Code for Information Interchange) text file format to accommodate future easier upgrades, if necessary. Also, to compensate for any increase in search time, the storage arrays were divided up into nine files so that there would be a faster access rate. ASCII text files are used to store precomputed plasma (_pa) and active (_act) insulin profiles as shown below for the four classes of insulin preparations catered for by AIDA online; Actrapid = short-acting; NPH + Lente = intermediate-acting; and UltraLente=long-acting insulin preparations; CAR = carbohydrate absorption profiles from the gut.

 Actrapid_act
 Actrapid_pa
 NPH_act
 NPH_pa
 Lente_act
 Lente_pa
 UltraLente_act
 UltraLente_pa
 CAR.

The amount of carbohydrates ingested in each meal determines the offset (0–80 grams) for fetching the appropriate absorption profile from the carbohydrate storage file. The data retrieved from the carbohydrate storage, along with the meal times, are used to calculate the glucose absorption profile over the 24-hour period. For each meal, the absorption of glucose into the blood stream from the gut is calculated over a period of several hours after ingestion. The type of insulin determines which of the eight insulin storage files to read, while the dosage determines the offset (0–40 IU) within that file. There are different forms of insulin injections offered as choices in the web-based simulator, as in PC AIDA v4: soluble short-acting insulin preparations (10 types), intermediate-acting preparations (19 types), long-acting preparations (4 types) [4], and a mixture of short- and intermediate-acting biphasic preparations (18 types).

Upon completion of the main computation process, over a simulated 24 hour period, the data is stored in six separate files from time 0 to 24 hours in preparation for graphing.

### 2.5. *Gnuplot* Graphing Program

AIDA online makes use of an open access graphical program, called *gnuplot*, to generate the AIDA online graphs in real time. The plots are displayed on a line graph with a frequency of 15 minute data points.

*Gnuplot* is a portable command-line driven graphing utility for Linux and many other platforms. The source code is freely distributed. It was originally created to allow scientists and students to visualise mathematical functions and data interactively, but, it has grown to support many noninteractive uses such as web scripting [33].

The six ASCII text files created by AIDA online and stored in the HTML area contain the *x*, *y* values for the graphical plots. Once these files have been closed, the simulator makes system calls that pass these files to the *gnuplot* graphing program, which takes in the *x*, *y* coordinates from the ASCII text data files and produces a graphic file.

In the original AIDA online version 1 development, *gnuplot* would produce a  .PPM (Portable Pixel Map) graphical file. However, web browsers did not support the  .PPM file type produced, so the simulator would make another system call to a separate program called ppmtogif to convert the  .PPM graphics file into a common  .GIF (Graphics Interchange Format) file supported by web browsers.

The  .GIF file is stored along with the data files in a temporary directory within the HTML area. This ensures that (i) the files can be accessed by the user for download which may assist data retrieval and (ii) the files are kept in the directory for approximately 6 hours before being deleted by an automated timed cronjob in order to save server hard disk space. With all the calculations and plot creations completed, the simulator displays its output in HTML format to the user's web browser. The HTML output automatically contains references to the newly created plots and data files for the user's browser to display.

The form page allows for a choice of which plots will be viewed. Once the simulator has finished its execution, the user also has the opportunity to download any of the plots as well as the actual data files [34].

The AIDA online website is designed so that multiple simulations can be run in succession. Each run of the simulator returns not only the graphs but also a new form already filled in with the data previously submitted. This allows the user to make changes to the input data based on their observations from the simulation graphs. When the user submits the new regimen data, new graphs are generated and a further data form is returned. Graphs containing the new information are overlaid on the previous plot. The user can therefore visually assess a change in the shape of the curves.

A key feature of the AIDA online diabetes simulation approach is the ability to visualise and compare graphical simulation results before and after a change in the regimen. This is achieved by the simulator submitting in the background not only the changed data and preferences but also references to the data files that were produced during the original simulation run. This is all done automatically, completely transparently for the user.

One of the ways in which user preferences are stored and communicated between simulation runs is via the use of hidden fields in the dynamically Perl-script generated HTML code. As shown in Figure 2(b), a series of parameters and variable values are recorded via this approach.

For a subsequent simulation, the simulator then goes through the same process as the original simulation run. However, this time, the previously stored data files are passed with the new data to the *gnuplot* graphing program. The new graphs will then contain values for the two different simulations. This allows the user to see the changes made from the previous run.

The present AIDA online simulator is set up to handle two separate data files at a time. The user can continue to submit changed data, but the new simulation will only contain the present and most recent previous simulation data. AIDA online was initially developed and run on a SuSE Linux 6.1 (SuSE Inc, CA, USA) world wide web server on a dual Pentium II/450 MHz (Intel, CA, USA) with 256 Mb RAM and 36 Gb DASD (Direct Access Storage Device). The web server software used was Apache 1.33 (Apache Group, CA, USA). 

The Apache web server development effort has aimed at creating a robust, commercial-grade, and freely-available source code implementation of an HTTP (HyperText Transfer Protocol) web server [35]. The Apache web server used on the Linux platform utilises a multithreading algorithm that allows multiple simultaneous processes to be run. For AIDA online, when the simulator is invoked, a Process Identification (PID) number is assigned. The files (graphics and data) are stored uniquely with the PID as part of the filename so even if multiple users from different parts of the world submit simulations at the same time, the simulations can all be processed separately. This explains how it is possible to run a diabetes simulation from, for example, Auckland, New Zealand, simultaneously with a simulation from Basel, Switzerland on a computer originally in Durham, North Carolina—and now in London, UK—all in a matter of seconds [36].

The AIDA online simulations can be run from any machine (PC, Apple Mac, Linux, Unix server, WebTV, smartphone, etc.) from anywhere in the world, provided the device has access to the internet and a graphical display.

There is also a free registration/announcement list that AIDA online users can subscribe to at the website (or by emailing subscribe@2aida.org) so that they can be immediately notified by email about any enhancements or modifications to the system as they become available worldwide. Furthermore, people can also follow developments with AIDA online on Facebook at https://www.facebook.com/www.2aida.org and/or at http://www.facebook.com/aida.diabetes.simulator1 or on Twitter at http://www.twitter.com/aida_diabetes.

The total elapsed time for a single simulation varies. There are basically two periods of delay between data being submitted by the user and the full results being presented back to the user. Firstly, the time for the simulator to be invoked and run depends on the amount of load on the server. However, the time used by the simulator is rarely more than 1 second. Secondly, there is a time delay for the transfer of the HTML page and graphics files back to the end user. The HTML (text only) file itself is only 9 kb (kilobytes) in size. By contrast, the standard simulation produces 17 kb, and the advanced (fluxes) simulation produces 25 kb of graphics data. While the total elapsed time for the user may initially have depended on the speed of the connection to the internet and geographical location, these file sizes are tiny compared with usual web transfers, so apparent simulator response times for users, wherever they are in the world, are generally very rapid (typically 1-2 seconds). There is also a http://www.sitemeter.com/ embedded graphic on the main AIDA online simulation web page which helps to monitor visitors running simulations and provides some anonymised insight into ongoing AIDA online usage. Loading this embedded graphic may delay the apparent simulation time—but the simulations are still incredibly quick, even via a home broadband connection.

Designed to be accessible and usable by anyone who desires to learn more about diabetes, AIDA online version 1 was initially made available on a Shodor Education Foundation web server in the Eastern United States (in North Carolina).

Shodor (http://www.shodor.org) is a North-Carolina-based research and education organisation dedicated to the advancement of science and maths education, specifically through the use of modelling and computers [37].

However, since then, with the development and release of AIDA online version 2 (AIDA online^2^), the main AIDA online facility was relocated by one of the authors (EDL) to London, England, and the web-based simulator has been updated further since, and merged in with the rest of the AIDA (http://www.2aida.org) website.

AIDA online^2^ has seen glycosylated haemoglobin (HbA_1c_) calculations as reported by Lehmann [38, 39] for AIDA v4—based on work by Nathan et al. [40]—incorporated into the web-based simulations, together with user-definable upper and lower normoglycaemic limits (bounds) which can be overlaid on the BG graph to assist users in identifying out-of-range high and low BG values.

The main AIDA online^2^ website currently resides on a shared web server with an Intel Xeon single core processor running at 2 GHz with 4 Gb of RAM. The current operating system is SuSE Linux Enterprise Server 10 (SuSE Inc, CA, USA).

### 2.6. Interacting with AIDA Online

Writing the program in Perl and then utilising a fast Linux server allows many users to access AIDA online simultaneously. The main educational utility seems to lie in AIDA online's ability to compare results from different simulation runs. For instance, by modifying the inputted insulin injections, users can learn to exert more control over the BG level of the simulated “virtual diabetic patient.” The working hypothesis underlying use of AIDA online is that, while modifying the input parameters, users—whether they be patients, carers, students, or HCPs—can also learn more about how to manage glucose concentrations in insulin-dependent diabetes mellitus (IDDM). The advantage of using multiple injection times and different insulin types becomes apparent as different regimens can be compared. Modifying carbohydrate inputs can also demonstrate the importance of maintaining a strict diet. Large swings in meal carbohydrate intakes result in obvious swings in BG levels that require adjustments to be made to insulin injections to correct the perturbations in the simulated patient. As has been highlighted previously by Blanchard et al., the advantage of having multiple preprogrammed simulated “patients” allows the user to see the effect of these changes on different body types that have various insulin sensitivities [41].

#### 2.6.1. Using AIDA Online to Perform Simulations

AIDA online is accessed directly at http://www.2aida.net or from the main AIDA website (http://www.2aida.org) via menu options on the left frame menu. Selecting “Online Simulation” takes the user through a short selection procedure, in which a case scenario may be chosen—if desired—and various preferences are determined. A summary of the key features of the AIDA online web-based diabetes simulator at http://www.2aida.net is as follows.

*Availability and Functionality*

- Accessible via the AIDA website at http://www.2aida.org or directly at http://www.2aida.net.
- Standard web browser software using mouse to navigate (by contrast to the downloadable standalone PC version of AIDA, also available at the website, which is DOS based and employs key and tab functions).
- Simulated blood glucose (BG) and plasma insulin levels are derived and displayed in graphical format.
- Carbohydrate intake and insulin injection data are also displayed graphically.
- Simulated glycosylated haemoglobin (HbA_1c_) value derived.
- Forty different case scenarios are available. Dedicated cases can also be created and simulated.
- Data entry forms allow the user to change various values and rerun simulations.

*Options*

- BG units: choose either mmol/L or mg/dL.
- Display: choose either “Standard” or “Advanced.”
- “Standard” displays the simulated BG and plasma insulin curves.
- “Advanced” displays the simulated BG and plasma insulin curves, together with graphs of the model's glucose fluxes (glucose absorption from the gut, glucose excretion from the kidneys, glucose utilisation in the periphery, and the net hepatic glucose balance: the production or utilisation of glucose by the liver).
- Insulin types utilised (when defining new case only): choose either “Standard” or “Premixed.”
- Display upper and lower bounds/limits (the user can define upper and lower normoglycaemic ranges to be indicated on BG graphs): choose either “Display bounds” or “Do not display bounds.”

*Data Entry Forms (user defined)*

- Six data entry points for the timing and quantity of carbohydrate intake over a 24 hour period.
- Insulin(s)—choose from a selection of short-acting and from a selection of intermediate- or long-acting preparations; up to four injections per day of each insulin type can be entered, along with the time and dose in units for each injection.
- Body weight is user defined (in kg or lb).
- Kidney function: renal glucose threshold can be “high,” “low,” or “normal” and renal function can be “reduced” or “normal.”
- Hepatic and/or peripheral insulin sensitivity may be normal or reduced.

**Figure 3:**
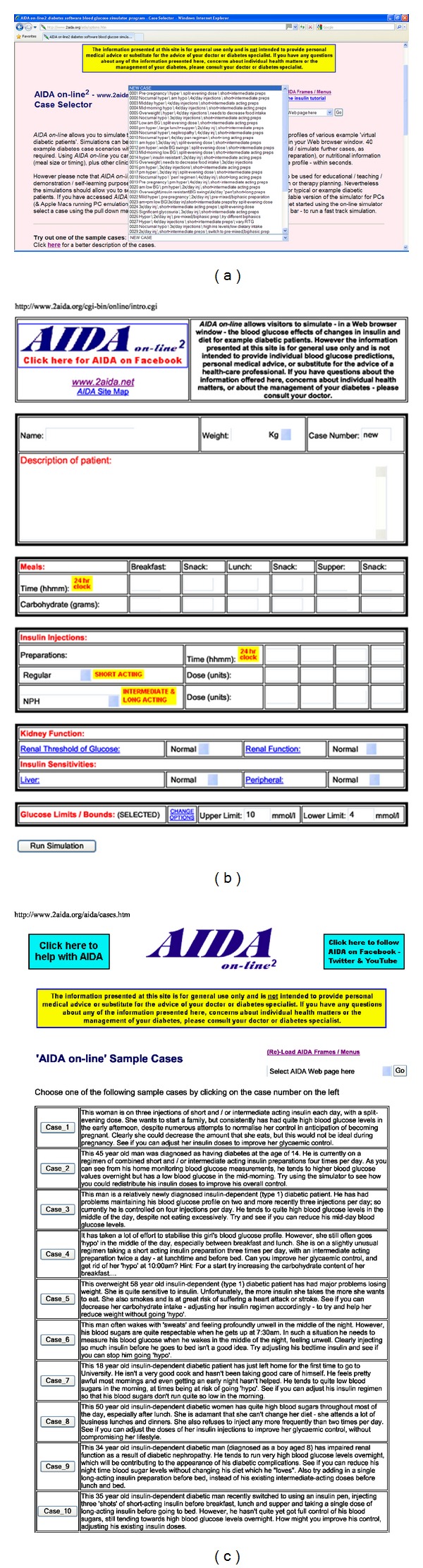
(a) AIDA online^2^ main options.htm Case Selector screen shown accessed via Internet Explorer on a Windows XP personal computer. The display shows how a list of summaries of each case scenario can be accessed via a pull-down menu. In this example, a NEW CASE is highlighted. (b) AIDA online^2^ intro.cgi dynamically generated HTML page created based on the NEW CASE selected shown in (a). As can be seen, all the fields are blank, allowing the user to create a new case from scratch. (c) AIDA online^2^ cases.htm fixed HTML web page giving further details of each of the first ten AIDA online case scenarios (imported from AIDA v4).

There are currently 40 different case scenarios to choose from as a starting point; each presents a virtual patient with IDDM on a given insulin regimen, experiencing real-life problems associated with BG control. Alternatively, as outlined above, a “new” case may be defined by the user (Figures 3(a) and 3(b)). If the user wishes to have further details about the case scenarios, these can be accessed on the options.htm page via the “*Click here for a better description of the cases*” link (see at the bottom of Figure 3(a)), which loads the cases.htm page (Figure 3(c)) giving full details of each case scenario.

The compartmental design of the AIDA model allows for a number of variables to provide a more tailored and realistic simulation. Hepatic and peripheral insulin sensitivities are modifiable into three categories (increased, normal, and reduced), as is the renal threshold of glucose (the level of BG at which sugar spills into the urine). Renal function is also modifiable into two levels, normal and reduced creatinine clearance (Table 1), which is then used for an estimate of the glomerular filtration rate (GFR).

The final two variables are probably of most interest to casual users, and these are the carbohydrate intake and insulin injections. Carbohydrates are input with a time of ingestion and a total number of grams. The insulin input allows for a time of injection, dosage, and type of insulin (short acting, intermediate acting, and/or long acting, with a variety of brand names to choose from). Additional input variables include the patient's name, weight, and definable limits of the recommended, allowed, normoglycaemic range.

**Figure 4:**
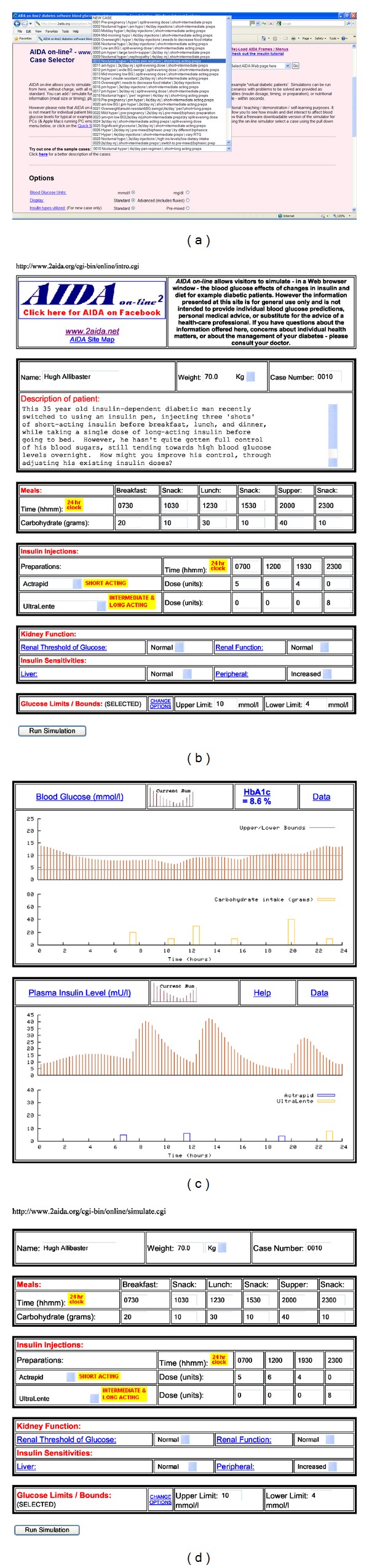
(a) AIDA online^2^ options.htm Case Selector screen showing Case Scenario 0010 highlighted. Options to select the “Blood Glucose Units” and “Standard” versus “Advanced (includes fluxes)” display are shown lower down the page. (b) AIDA online^2^ dynamically-generated HTML web page, produced by intro.cgi Perl script, populated with information and data for case scenario number 0010 (Hugh Allibaster). Clicking on the (Run Simulation) button at the bottom of the web page will submit the data shown to the simulate.cgi script to run the simulation engine and generate a graphical simulation (as shown in Figure 4(c)). (c) Baseline simulation using AIDA online^2^ with data shown in Figure 4(b) for Hugh Allibaster (case scenario 0010). Top panel: Blood glucose (BG) level and carbohydrate intake over a 24 hour period. User definable normoglycaemic ranges (“Upper/Lower Bounds”: 4–10 mmol/L [72–180 mg/dL]) are shown superimposed. Lower panel: Plasma insulin level and injections of Actrapid (short-acting) and UltraLente (long-acting) insulins. The HbA_1c_ value (8.6%) gives an indication—for educational purposes—of the glycosylated haemoglobin level predicted by the AIDA model, if the current glycaemic control were to be maintained in the medium- to long-term. (d) Scrolling down the simulate.cgi page shown in Figure 4(c) yields a dynamically-generated HTML data entry form populated with the data used for the simulation, which—for a baseline (initial) simulation—will be identical to that shown in Figure 4(b). The user can now change any of the data values and run a further simulation by clicking on the (Run Simulation) button at the bottom of the screen.

Having selected a case scenario via the pull-down menu on the options.htm page (Figure 4(a)) or via the cases.htm page (Figure 3(c)) and/or indicated preferred options, the (Continue) button is clicked and a Perl script (intro.cgi) stored in the cgi-bin area is invoked. A new dynamically generated page is loaded, giving a description of the selected case (see Figure 4(b)). Data entry forms are displayed, with prestored entries shown for carbohydrate intake (amount and time) and insulin dosage (type, amount, and time), according to the selected case scenario. If the “New Case” option has been selected, then this form is presented blank (Figure 3(b)). There are up to six data entry times for carbohydrate intake (for breakfast, midmorning snack, lunch, afternoon snack, supper, and bedtime snack) and up to four data entry times for each of two insulin types, to cover a 24-hour period, which enables a wide range of insulin regimens to be accommodated.

Preselected data pertaining to the chosen case scenario can be modified at this stage, if desired, prior to running the simulation.

A number of other parameters can also be changed, as outlined previously and inTable 1, and these help in further defining the virtual patient's clinical situation.

When all of the data entries have been made and the (Run Simulation) button is clicked (Figure 4(b)), then the simulation is performed in real time.

If the online simulator is accessed directly from the popup window that is automatically generated when the AIDA website is loaded or if “Quick Simulation” is selected from the left frame menu, then the user is taken straight into a preselected case example (case number 0001, “Joy Wilson” in the AIDA online case scenario database), in which the baseline simulation has already been performed (see http://www.2aida.org/aida/example.htm). This route provides an ideal introduction to AIDA online for first time users.

Once a simulation is run, a new page is loaded, which gives the simulated BG and plasma insulin levels for the 24-hour period (Figure 4(c)). The first panel shows BG fluctuations plotted against time, with a dotted line indicating preferred limits (normoglycaemic range), if this option was selected. Directly below, on the same chart, carbohydrate intake is quantitatively indicated across the 24-hour period. The bars at the bottom of the graph, showing the carbohydrate intake, help the user to visually line up the changes in the BG graph based on carbohydrate intake.

The second panel shows simulated plasma insulin levels over the same 24-hour period; injected insulin doses are indicated below, on the same chart. Once again, the bars below the plasma insulin levels are similar to the carbohydrate graph, except showing insulin injections.

This graphical presentation of both user-defined and simulated data clearly illustrates the relationship between meal times, carbohydrate consumption, and associated BG excursions, insulin injections, and plasma insulin levels. By the same token, the graphs provide an uncomplicated presentation of the characteristics of various insulin types, particularly in terms of their action profiles.

**Figure 5:**
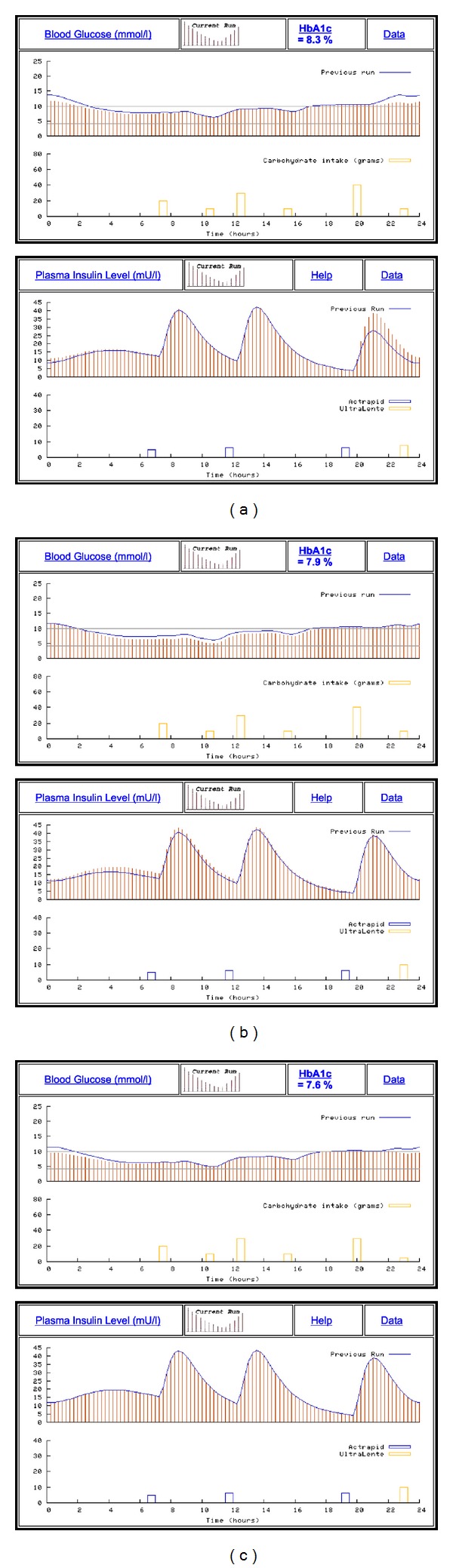
(a) AIDA online^2^ simulation of the case shown in Figure 4(c) in which the evening (7:30 pm) Actrapid dose has been increased by 2 units from 4 units to 6 units. The current simulation run is shown as the vertical red lines, with the previous run shown for comparison as the solid blue lines. The higher plasma insulin peak in the evening and the reduction in blood glucose (BG) overnight are clear to see, although the BG profile still remains elevated compared with the user defined normoglycaemic range. The glycosylated haemoglobin (HbA_1c_) level—if this control were maintained in the medium-to-long-term—is expected to improve from 8.6% (Figure 4(c)) to 8.3%. (b) AIDA online^2^ simulation of the case shown in (a) in which the evening (11 pm) Ultralente dose has been increased by 2 units from 8 units to 10 units, resulting in some further improvement in the blood glucose (BG) profile, with an expected glycosylated haemoglobin (HbA_1c_) of 7.9% (improved from 8.3% in (a)). The current simulation run is shown as the vertical red lines, with the previous run shown for comparison as the solid blue lines. (c) AIDA online^2^ simulation of the case shown in (b) in which the bedtime snack has been decreased by 5 grams from 10 grams to 5 grams and supper has been decreased by 10 grams from 40 grams to 30 grams (in one simulation). This results in further improvement in the simulated blood glucose profile with a predicted glycosylated haemoglobin (HbA_1c_) of 7.6%. Once again, the current simulation run is shown as the vertical red lines, with the previous run shown for comparison as the solid blue lines.

A key feature of AIDA online is the facility that enables one simulation to be compared with the previous. Thus, the HTML data forms are reproduced on the same page below the simulation panels, allowing the user to make any number of changes to the input data (Figure 4(d)). Re-running the simulation derives similar graphs; however, the BG and plasma insulin levels from the previous simulation are also shown. Taken together, the two panels illustrate how various features of the insulin-diet regimen interact, and the user can observe and identify specific effects arising from any changes made prior to rerunning the simulation. In addition, the computed glycosylated haemoglobin (HbA_1c_) value gives a predicted indication of overall BG control, and this helps to guide the learning process, especially when simulations are rerun repeatedly (Figures 5(a), 5(b), and 5(c)).

**Figure 6:**
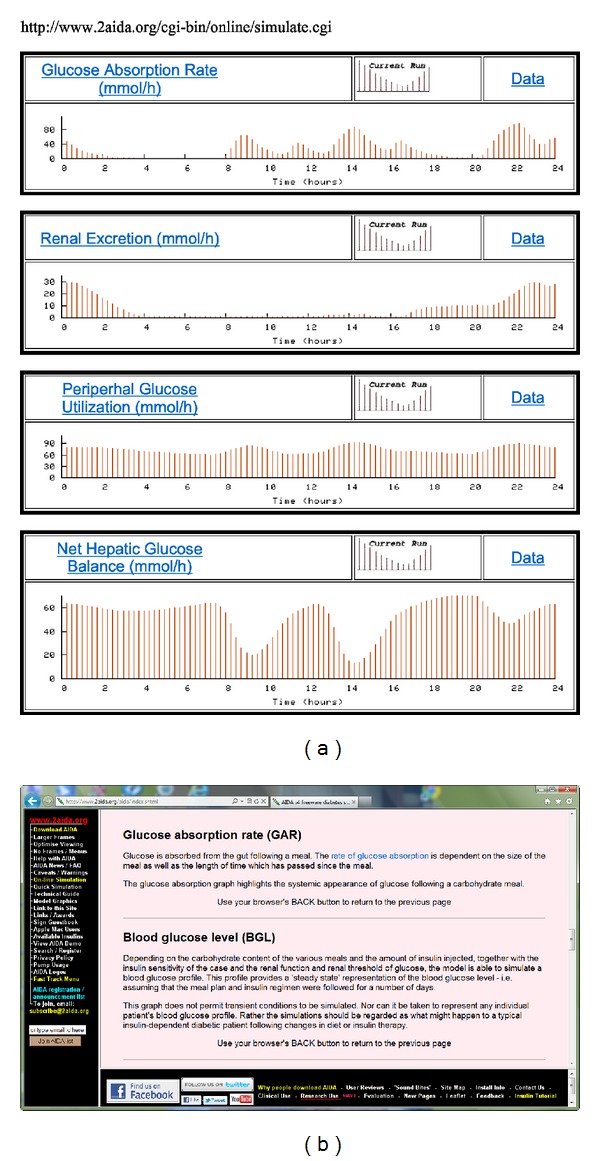
(a) Advanced Display option showing fluxes for the baseline simulation from Figure 4(c). “Glucose Absorption Rate” shows glucose absorption following carbohydrate ingestion in meals; “Renal Excretion” shows the passage of glucose into the urine, above the renal threshold of glucose; “Peripheral Glucose Utilization” demonstrates the utilization of glucose in the periphery, while “Net Hepatic Glucose Balance” shows the production or utilization of glucose by the liver. (b) Screenshot from AIDA online^2^ showing two of the entries in the online glossary—directly accessible at http://www.2aida.org/glossary—which is linked to the online simulator output. In this way, users can click on HTML links to obtain explanations about terms they do not understand or concepts with which they are unfamiliar.

The user has the option to view several additional graphs that help explain how the BG simulations are derived. These additional graphs include glucose absorption rate, renal excretion of glucose into the urine, peripheral glucose utilization, and net hepatic glucose balance (Figure 6(a)), which are “fluxes” in the original standalone PC AIDA v4 program.

Throughout the simulation web pages are HTML links to an online glossary (Figure 6(b)), which gives clear but concise explanations of basic terms—for the newcomer—such as BG level, plasma insulin level, and HbA_1c_. So, clicking on underlined links shown, for instance, in Figures 4(a)–4(d), 5(a)–5(c), and 6(a) all link to the online glossary for further explanations. More advanced scientific concepts associated with the model used in the simulations are also described. Thus, for those users already familiar with the relevant basic concepts, glucose flux options are described, and HTML links provide access to graphical representations of the model of glucose utilisation that is used (also available by selecting “Model Graphics” from the left frame menu). Furthermore, there are links to graphs showing insulin absorption and elimination for various types of insulins covered by the simulator. Additional detailed technical information about the AIDA model is also freely available to users at the AIDA website, accessible directly at http://www.2aida.org/technical. A summary of how AIDA online works is also explained via a link at the bottom of the online simulation web page.

Employing standard web-browser technology, AIDA online is easy to use, and simulator response times are generally very rapid.

#### 2.6.2. Caveats and Warnings

It is emphasised that the model upon which AIDA online is based is not sufficiently accurate for individual patient BG prediction or therapy planning [15, 42, 43]. The user is therefore reminded that the simulation approach is suitable only as an educational tool and not for generating individual therapeutic advice or treatment planning. The general caveats displayed at the AIDA website can be seen at the top of Figures 3(a) and 3(c), and the AIDA online specific caveats can be seen top right in Figure 3(b).

#### 2.6.3. Applications of AIDA Online

Current applications of AIDA online are numerous and wideranging, as exemplified through the variety of user-generated feedback received which has on the whole been very positive. Independent user reviews of AIDA/AIDA online have been published previously elsewhere [44, 45], and user feedback can also be found at the AIDA website at http://www.2aida.org/aida/online-reviews.htm and is referred to in the results section of this paper.

To make the most effective use of AIDA online, it may help for the web-based simulator to be incorporated into structured learning materials. One way in which the simulation approach may be tied in with “static” textual educational material to effectively enhance the learning experience is illustrated at the AIDA website by the interactive Diabetes/Insulin Tutorial [46]. However, use of AIDA online is not limited to personal or individual distance learning via the internet; it also has the potential to be used successfully in a variety of settings, including group education sessions [44].

As has been highlighted by DeWolf [47], computer-assisted education has the potential to accommodate different *speeds* of learning. Some diabetes education sessions have often been provided to groups of patients/students and have necessarily been administered at the *average* learning speed of the group. However, computers can also offer education directly to individuals, allowing individuals to control the pace of their own learning and at the same time receive immediate feedback. AIDA online can service both group and individual teaching sessions [47].

### 2.7. Technical Issues

There have been some technical issues with AIDA online, particularly in the early years. The original web-based simulator made use of a program called ppmtogif. This converted  .PPM format files generated by *gnuplot*, which could not be viewed by browsers, into  .GIF format files, which are very well supported by web browsers.

While  .GIF files have been very widely used on the internet, there were unfortunately some issues about programs which produce  .GIF files online needing to be licenced prior to use. The licence fee, apparently, cost US$5,000, and therefore, the ppmtogif program could no longer be provided with a GNU public licence [48].

Given this, when AIDA online was moved from North Carolina to London, UK  .GIF files could no longer be generated in realtime by the web-based simulator. As a result, the initial release of AIDA online version 2 (AIDA online^2^) was modified to make use of  .JPG (Joint Photographic Experts Group) and  .PNG (Portable Network Graphics) image files, avoiding any problems with dynamically generating  .GIF files. Subsequent versions of AIDA online^2^ have been modified further to just concentrate on  .PNG format graphics, which actually reduce the file sizes, contributing further to the apparent speed of AIDA online^2^ (Table 2).

## 3. Results

The conversion of the AIDA v4 Pascal program from the DOS-based standalone, downloadable version to Perl has been successful, and AIDA online has been accessible via http://www.2aida.net for a number of years.

**Figure 7:**
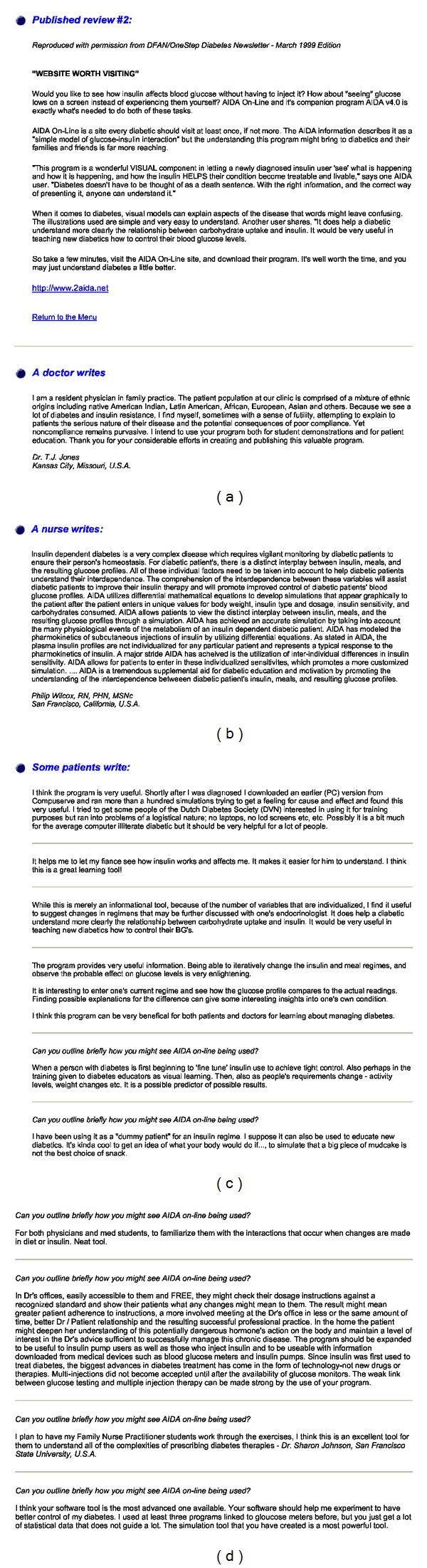
Independent user reviews of AIDA online, reproduced from the AIDA online website (http://www.2aida.org/aida/online-reviews.htm).

The purpose of developing a world wide web accessible glucose-insulin simulator has been to provide an educational opportunity for as many people as possible (patients with diabetes, their relatives, students, and HCPs). In this respect, AIDA online has exceeded all expectations. A range of user comments/reviews can be found in Figures 7(a), 7(b), 7(c), and 7(d).

AIDA online^beta^ first went “live” on the internet for beta testing in December 1997. AIDA online version 1 was formally launched in August 1998, following 8 months of extensive beta testing by the authors and others.

AIDA online has been thoroughly tested with a range of web browsers and works well with Microsoft Internet Explorer, Mozilla Firefox, Google Chrome, and Apple Safari, as well as with Netscape Navigator (in earlier years).

**Figure 8:**
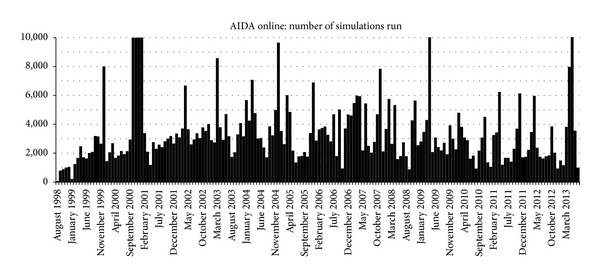
Usage of AIDA online showing the number of simulations run per month versus time. (a) January 1999—server hosting “AIDA online” version 1 crashed, and therefore the facility was unavailable for much of the month. (b) October 2000 to January 2001—simulated blood glucose data harvested from AIDA online by researchers at NASA for training and testing an insulin-dosage adjustment decision support prototype. Over 68,000 simulations were run during this time. Further information available at http://www.2aida.org/nasa.

Since records began, over 637,500 diabetes simulations have been run at AIDA online (Figure 8). Since AIDA online was relocated to a server in London, UK, visitors to the website have been logged from over 115 countries, including (in alphabetical order): Andorra, Argentina, Armenia, Aruba, Australia, Austria, Bahrain, Belarus, Belgium, Bermuda, Bhutan, Bolivia, Bosnia-Herzegovina, Brazil, Brunei Darussalam, Bulgaria, Canada, Chile, China, Christmas Island, Cocos (Keeling) Islands, Colombia, Comoros, Costa Rica, Croatia (Hrvatska), Cuba, Cyprus, Czech Republic, Denmark, Dominican Republic, Ecuador, Egypt, Estonia, Ethiopia, Faroe Islands, Fiji, Finland, France, French Polynesia, Georgia, Germany, Gibraltar, Greece, Guam, Guatemala, Hong Kong, Hungary, Iceland, India, Indonesia, Iran, Ireland, Israel, Italy, Jamaica, Japan, Jordan, Kazakhstan, Kuwait, Latvia, Lebanon, Lesotho, Lithuania, Luxembourg, Macau, Macedonia (Former Yugoslav Republic), Malaysia, Malta, Mauritius, Mexico, Moldova, Morocco, Nepal, Netherlands, New Caledonia, New Zealand (Aotearoa), Nicaragua, Nigeria, Norway, Oman, Pakistan, Panama, Papua New Guinea, Paraguay, Peru, Philippines, Poland, Portugal, Qatar, Romania, Russia/Russian Federation, Samoa, San Marino, Saudi Arabia, Singapore, Slovakia/Slovak Republic, Slovenia, South Africa, South Korea, Spain, Sri Lanka, Sweden, Switzerland, Taiwan, Thailand, Tonga, Trinidad and Tobago, Turkey, USSR (former), Ukraine, United Arab Emirates, United Kingdom, United States of America, Uruguay, Venezuela, Yemen, and Yugoslavia (former), as well as from nonprofit organizations, unknown IP addresses, US commercial, educational, government and military IP addresses, and dot.net and dot.arpa (old Arpanet) addresses. Independent comments about the simulations have been very encouraging [44, 49–51]. On average, more than 3,200 simulations have been run each month at AIDA online (in 2011–2013).

**Figure 9:**
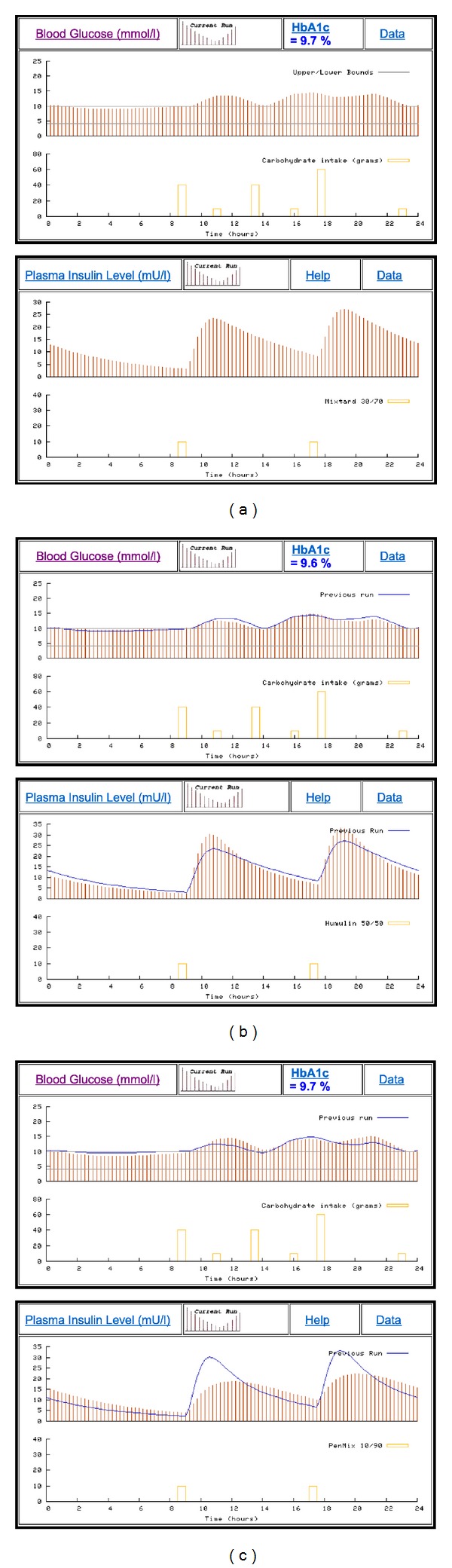
(a) Gives a baseline simulation for case scenario 0026 in the AIDA online database, showing a premixed (biphasic) insulin injection regimen with Mixtard 30/70 being injected twice a day. Mixtard 30/70 is a premixed mixture of 30% short-acting insulin and 70% intermediate-acting insulin. (b) Demonstrates the effect of switching the insulin type from Mixtard 30/70 shown in (a) to Humulin 50/50 (50% short-acting and 50% intermediate-acting insulin) with proportionally more short-acting insulin. The current simulation run is shown as the vertical red lines, with the previous run shown for comparison as the solid blue lines. (c) Demonstrates the effect of switching the insulin type from Humulin 50/50 shown in (b) to PenMix 10/90 (10% short-acting and 90% intermediate-acting insulin) with proportionally more intermediate-acting insulin. The current simulation run is shown as the vertical red lines, with the previous run shown for comparison as the solid blue lines.

Figure 9 shows a series of simulations using case scenario 0026 in the AIDA online database. The case details for this scenario record that “*This young woman is on a twice daily insulin regimen, injecting a biphasic preparation which has a premixed 30% to 70% ratio of short versus intermediate acting insulin. While this does not permit quite as much flexibility in selecting a dose*—*it does save on having to mix insulin in the syringe. Use the simulator to see what would happen if you switched this woman onto other biphasic preparations with, say, premixed 10/90, 20/80, 40/60, or 50/50 percent constituents…*”

The simulations given in Figures 9(a), 9(b), and 9(c) demonstrate the usage of AIDA online with premixed (biphasic) insulin injections containing different proportions of short-acting and intermediate-acting insulin preparations.

## 4. Discussion

The work overviewed in this paper shows how it has been possible to make use of the internet not just as a static repository of information but also as an interactive medium to perform quite complex dynamic simulations in a clinically useful manner. Translating the original PC AIDA v4 Pascal source code into Perl and then utilising a fast web server have allowed many users to access AIDA online simultaneously. The authors are not aware of any diabetes simulation facility as sophisticated as AIDA online that is available anywhere else on the internet.

The main educational utility of AIDA online lies in the ability to rapidly compare results from different diabetes simulations. By modifying the simulated insulin injections, users can learn to exert more control over the BG level of the simulated patient. For instance, possible advantages of using multiple injections and different insulin types can become apparent as different therapeutic combinations are compared. Modifying carbohydrate inputs can also demonstrate the important contribution of diet to BG control. In this respect, large swings in meal carbohydrate intake result in obvious swings in the BG level, which requires insulin injections to be adjusted for the simulated virtual diabetic patient. Having multiple, prestored, example “patients” allows users to see the effects of these changes on different patients with different sensitivities to insulin.

The hope is that patients, their relatives, and students can learn how to balance insulin and diet in diabetes by modifying the simulations. The concept underlying this diabetes simulation approach is that patients with diabetes may also improve their ability to actually manage their own diabetes by experimenting in this way. Clearly, this hypothesis remains to be tested in a clinical randomised controlled trial (RCT) setting [52, 53]. However, it is hoped that the relatively widespread use—and widespread availability—of the AIDA PC and AIDA online diabetes simulation approaches will encourage the undertaking of such clinical trials.

There are potential advantages and limitations to both the downloadable and web-based versions of AIDA. However, in reality, the two simulators complement each other remarkably well. One benefit of AIDA online has been to allow visitors to the AIDA websites to try out the simulator—on the web—before downloading a copy to use locally on their PC/Mac. Although not specifically studied, the working hypothesis for this complementary approach is that if people like what they see using AIDA online, they may be more likely to download the standalone (PC) version for further use locally on their home/office computer.

AIDA online extends the concept of the downloadable AIDA PC software by making the simulator accessible via the world wide web from any device (e.g., UNIX based, Apple Mac, or network server computer), smartphone, or WebTV anywhere in the world provided it has internet access and a graphical display. There is no longer a need to have a PC nor to download and install software on a local machine. Furthermore, upgrades to AIDA online require only one version of the program to be changed, and these changes then automatically become accessible to anyone from anywhere in the world. In addition, many users have asked for a windows-based, mouse-controlled “point and click” version of AIDA [54]. AIDA online provides just such an easy to use simulator with a standard user interface accessible in a familiar web browser format.

### 4.1. Limitations

There are some obvious limitations to AIDA and the underlying model. Physical activity is currently not addressed and assumed to be constant over the course of the steady-state simulation period. It is recognised that different carbohydrate types are absorbed at different rates by the body, but for the purpose of the model, all carbohydrates are considered equal. In addition, although there are modifiable variables for medical conditions, such as renal disease, they are not able to be specifically tailored to exactly recreate a specific patient's condition. Some of this is done intentionally, to keep patients from feeling that they can model their own particular treatment regimen and expect the results obtained to exactly mimic their own BG values. However, the more variables (parameters) included in a model, the more complex it becomes.

One of the not-so-obvious limitations of the model results not from the model itself, but from the patient's own day to day variances, be it diet, insulin dosing, BG data collection, or otherwise, which can affect their own BG readings. Many of these variables (such as exercise, stress levels, or food intake) can change minimally and not be noted by the patient. This was especially notable during the validation process for the original AIDA model [15].

These and other limitations may help to explain why there has been difficulty in applying these models to individual patient therapy planning in clinical practice. However, this does not negate the educational benefit of allowing users to see how carbohydrate intake and insulin usage will affect BG levels on an ongoing basis throughout the day and allow users the opportunity to see how plasma insulin and BG levels interact over a 24-hour time period.

Other limitations of the AIDA online approach relate to the fact that a user clearly needs continuous internet access to use the web-based simulator. In earlier times, this was fine for those in academic/work establishments with continuous web access and for those with unmetered access, for example, in first world countries. However, broadband/dial-up usage does remain an issue for those who still pay by the minute for internet access, for example, via dial-up in certain parts of the world, or who only have time-limited satellite-based internet access, for instance, in some parts of Africa. As there are still many parts of the world with intermittent/no web access, it is recognised that a web-based application like AIDA online will be only of limited benefit in those places, and perhaps the downloadable standalone PC version of the simulator will actually remain of more use in those areas.

Interestingly, the centralised versus distributed computing idea of AIDA online versus AIDA v4 revisits a number of early discussions about PCs versus central servers in years gone past. Things seem to have gone full circle now with “cloud” computing.

### 4.2. Other Considerations

At present—with its current level of usage—AIDA online does not require a dedicated server. However, potentially one web-based server could have difficulty supporting the extent of usage which has been reported with the downloadable AIDA PC software. For instance, a detailed survey of AIDA v4 users has revealed that 16,790 simulations were run by 200 users [54]. Over a similar period—around the same time—over 16,700 downloads of the AIDA PC software were logged at the main AIDA website. If the survey usage was typical for most AIDA v4 PC downloads, this would suggest that in the region of 16,700 × (16,790/200) = 1,400,000 simulations might have been run on these 16,700 downloaded copies of the PC software.

From past experience, 2,600 AIDA online simulations run per month account for approximately 610 Mb of website data traffic each month (Dr. E. D. Lehmann, personal communication).

Over a comparable two-year period, based on the AIDA v4 PC survey data [54], AIDA online would have needed to support roughly 23 times as many simulations (approximately 58,300 simulations/month) to run as many simulations as are estimated to have been executed by the AIDA PC software. This would equate to approximately 14 Gb of data traffic per month (23 × 610 Mb). While these are only estimates, 14 Gb of data transfer per month just for AIDA online would be perfectly feasible to support in internet/web/computing terms. However, together with static HTML traffic from the Diabetes/Insulin Tutorial and AIDA online glossary and PC AIDA v4 software downloads which independently account for 4-5 Gb of data traffic each month (Dr. E. D. Lehmann, personal communication), careful consideration may need to be given to the web hosting company used to permit such an ongoing level of sustained activity.

Furthermore, as an unfunded, not-for-profit, and noncommercial venture, the cost of a dedicated server may be hard to justify just to run AIDA online. Therefore, the web-based simulator is currently hosted on a *shared* server, which substantially reduces the hosting cost.

However, in order to render the graphical simulations, AIDA online relies on local access on the same server to the *gnuplot* program. Access to *gnuplot* is not always included as standard on shared Linux servers; *gnuplot* needs to be compiled on the server for installation, which requires root access. This clearly is possible with a dedicated server, where root access and installation are feasible, but on shared servers, this necessitates the support and cooperation of the web host. Fortunately, *gnuplot* is accessible as part of the “Ruby on Rails” open-source web framework (http://www.rubyonrails.org), which appears to be supported on more shared Linux servers, and in this way, it seems to be possible to find web-hosting companies that will support longer-term access to *gnuplot* even on shared Linux server platforms.

Clearly, at times, the number of simulations run at AIDA online will be high, for example, when people are running diabetes education classes making use of the web-based simulator. At other times, the load on the server will be much lower. It is difficult to predict these surges in usage, but to maintain its position as a well-established, much used web-resource, the AIDA online simulation facility needs to be able to cater for peak demand.

With only one web server running the online simulator if this server goes down, there would be no online diabetes simulations. One solution would be to make use of “mirror sites,” but in the early days of AIDA online, cost may have been an issue. However, web hosting costs have fallen dramatically since, so hosting multiple AIDA online simulators on different shared web servers now becomes more practical.

This consideration is of importance particularly if the intention is to encourage third party websites to link to AIDA online and the Diabetes/Insulin Tutorial [46]. For such links, it becomes imperative to offer a good “service,” because if AIDA online becomes unavailable, then the service at the other websites will also be restricted. Clearly, other website owners will not wish to make a commitment to using interactive educational simulations, however good, unless they can be assured that the service will be reliable.

Therefore, in the longer term, if AIDA online is to be used even more extensively, a distributed network of mirrored low-cost shared web server accounts might be an appropriate way to proceed. This could be less costly and more flexible than one large dedicated (expensive) server and could help to ensure that the web-based simulator would remain available irrespective of local geographic server issues. One might even consider porting AIDA online to a “cloud” computing cluster, so that usage of the simulator could be scalable and increased, as required by user demand.

Obviously, keeping AIDA online working on multiple servers would raise issues about longer-term maintenance, aside from cost. However, it seems preferable to keep AIDA online operating on a number of separate, distributed web-servers—in different geographical locations—to ensure the web-based simulations can continue to operate irrespective of local issues with any one single web server. For this reason, the process of mirroring AIDA online^2^ has started, and one of the authors (EDL) has set up a mirror server for AIDA online^2^ in California, USA.

### 4.3. Future Developments

The authors believe that the full potential of such web-based interactive educational diabetes simulators is yet to be fully realised. As a result, AIDA online and the AIDA website form part of an ongoing development. A number of simple refinements are already planned, but with collaboration and further resources, the novel web-based simulator could be incorporated into a wider range of diabetes educational resources in a number of innovative ways and make an even more significant contribution to the rapidly evolving field of web-based learning.

#### 4.3.1. New Insulins

Recent years have seen the successful introduction of a number of insulin analogues with markedly different action profiles compared with earlier insulin preparations. As these types of insulin are becoming ever more popular with patients and HCPs alike, it will be pertinent to adapt AIDA online to accommodate use of these newer insulins. Such work has been done for AIDA v4 [55–58], and now the concept needs to be ported to an updated release of AIDA online^3^ (version 3).

#### 4.3.2. Insulin Pump Therapy

Insulin pumps are increasingly being recognised as an effective way to provide physiological insulin replacement in people with IDDM. As it stands, the AIDA model does not explicitly cater for continuous subcutaneous insulin delivery; however, user feedback has indicated that useful insight can still be gained from the software by using basal levels of insulin and adding in insulin boluses, Ways in which AIDA and AIDA online can be applied for insulin pump simulations, derived from: http://www.2aida.org/pump, are as follows.

To set up a near “basal” level of insulin administration, it is possible to make two entries within AIDA online using a long-acting insulin preparation (e.g. Ultratard, Humulin-U, or Ultralente). These two “injections” need to be set 12 hours apart (say in the morning and evening).

As AIDA and AIDA online are currently limited to 4 insulin administrations (injections) per day, if a user can make one of these long-acting entry times correspond to a meal (and therefore a bolus), that will permit 3 boluses to be given to AIDA / AIDA online, at other times. For example, if a user would like to simulate a basal regimen with 10 units/day, with AIDA or AIDA online the user could enter 4 units of Ultratard in the morning (at 7 am) and 6 units in the evening (at 7 pm). This would give a background insulin profile that is slightly elevated in the morning, which might be fairly typical for a patient who does not have too much of a blood glucose (BG) rise in the morning.

If a user would like to simulate a bigger morning BG rise (e.g., as in the “dawn phenomenon”), then with AIDA/AIDA online, it is possible to use a slightly more lopsided ratio. For instance, for the 10 units/day Ultratard example given above, a user could split the dose to 3 units in the morning and 7 units in the evening, for the basal background. However the user would need to be careful to not split the basal dose, say 1 unit am and 9 units pm, since the simulator will need some morning long-acting insulin to help flatten out the basal curve in the afternoon.

For the bolus doses, AIDA/AIDA online will allow users to give the simulator up to 4 boluses (injections) of short-acting (regular) insulin, although one limitation is that 2 of these will need to be at the same times as the basal doses. Nevertheless, by giving the simulator a basal and bolus dose together (for instance in the morning), this will still leave users with at least 2 more (and possibly 3) boluses to “play with” later in the day.

It is hoped that in this way—by experimenting with different basal and bolus regimens—users might be able to learn a bit more about balancing insulin and diet in diabetes, even with an insulin pump.

A version of the simulator software which would enable pump regimens to be more completely simulated could be planned. Ultimately, it is hoped that AIDA online may be usefully incorporated into insulin pump training programmes, as a means of illustrating some of the principles of physiological insulin therapy.

#### 4.3.3. Enhancing Structured Education Resources

Flexible insulin therapy is increasingly being recognised as an effective approach to insulin treatment. The Diabetes Control and Complications Trial (DCCT) [59] demonstrated the long-term benefits of intensive insulin treatment; however, the increased risk of hypoglycaemia and increased staff resources used in the DCCT have perhaps limited the adoption of this approach even more widely. However, more recent evidence suggests that appropriate training in insulin adjustment can improve HbA_1c_ without significantly increasing the risk of severe hypoglycaemia [60, 61]. Diabetes self-management skills training enables patients to adapt insulin doses on a daily basis, in order to accommodate increased dietary freedom. This approach originated in Germany but is now widely adopted in many countries. The Dose Adjustment For Normal Eating (DAFNE) trial in the UK [60] has led to the approach receiving increased support, and this educational material is even being adapted for school-age children [62]. Modular outpatient education for flexible insulin treatment adapted for pregnancy has also been shown to improve pregnancy outcome [63].

Web-based diabetes simulators, such as AIDA online, could conceivably be used in such skills-based training programmes to aid understanding of the relationships between insulin dose/timing and meal content/timing. Further refinement of the software to allow for the effects of exercise and/or illness or stress would obviously confer even greater potential. Additionally, the use of web-based resources in training programmes such as those used in the DAFNE approach may help to address the perceived problems associated with staffing levels, time, and resources.

The interactive educational Diabetes/Insulin Tutorial at the AIDA website [46] demonstrates how AIDA online can be integrated with “static” informational material to provide an even more engaging educational resource. It is envisaged that this concept could be expanded further. In particular, AIDA online could potentially be integrated with teaching materials for various different specific audiences, with the text and format of the informational material being tailored appropriately to the target audience. For example, AIDA online could be used in structured training programmes for diabetes HCPs, educators, and students. Specific educational materials could be directed towards various patient populations: teenagers, young women considering pregnancy, patients with low health literacy, patients with noninsulin dependent diabetes mellitus (NIDDM) beginning insulin treatment, and so on. An interactive learning game for school-age children may also be of tremendous value. While it is noted that the AIDA simulator model is based on data for adults with an absolute insulin deficiency, it can still be used effectively to demonstrate the *principles* of insulin adjustment even if some endogenous insulin secretion is still occurring.

AIDA online can also be run across the internet from third-party websites, in a separate “pop-up” or new web browser window. This increases the possibilities for further collaborative intelligent use of the simulator in web-based diabetes education resources.

In particular, there are large numbers of static graphical/fixed textual diabetes educational resources on the internet but not so many online diabetes simulators. In Reed and Lehmann [46], the concept is developed of one simulation engine being able to service a number of educational websites. So, for instance, not all diabetes educational websites need to have the complexity of running AIDA online locally, with (common gateway interface) cgi-bin programs/scripts and *gnuplot* available. With the interconnected web, each educational diabetes resource does not need to run its own copy of the simulator locally.

Therefore, a Diabetes/Insulin Tutorial has been developed at http://www.2aida.info and integrated with AIDA online, which can also now be accessed at other websites—for example http://medweb.bham.ac.uk/easdec/aidadevelopment/tutorial.htm—and the tutorial has even been translated into other languages—for example, Spanish (“Tutorial sobre Insulina y Diabetes”) at http://www.um.es/grupo-cirrosisInsulintutorial/tutorial87.htm Further collaborative website developments like this are expected.

Refinement of the simulator to incorporate the effects of exercise, illness, and stress would be useful. Additional benefits may also be derived from adding in functions to allow for some variation in the glycaemic index of carbohydrates consumed.

The AIDA model thus far has been centred on people with IDDM and focuses on the diet and insulin interactions instituted by the patient/student. Of necessity, many of the interactions have been simplified. This is the nature of modelling to present as simple and as accurate a version of an event as possible.

The validation work performed on the original downloadable AIDA software precluded using this method as a treatment planner but was found to be sufficiently accurate to warrant use as an educational tool [15]. The same issues apply to AIDA online, and this fact is extensively noted at the AIDA website to try and prevent patients from directly applying data from AIDA online simulations to their personal treatment plans (e.g. see caveats in Figures 3(a) and 3(b)).

By focusing predominantly on insulin therapy and carbohydrate intake, while unobtrusively accounting for some of the significant medical modifiers, AIDA online allows users to experiment with various regimens and see how they may affect the overall glucose-insulin balance.

This is done by providing a relatively simple interface for the user to input/modify times and amount of carbohydrate intake, as well as insulin dosages, and providing a wide variety of insulin types to choose from. The simulation can then be run in either a basic (standard) or advanced mode, with the difference being in the number and type of graphs that are displayed.

As part of future work, it is intended to increase the number of AIDA online case scenarios available on the web. Already, three extra cases have been generated, which are accessible directly at http://www.2aida.org/aida/fast-track8.htm, http://www.2aida.org/aida/fast-track1.htm, and http://www.2aida.org/aida/fast-track14.htm These just now remain to be included in the main AIDA online case scenario database. Additional case scenarios will be added, particularly when new cases are generated for the lispro/glargine release of AIDA v4 (v4.5c) and once the new insulin analogues (lispro and glargine [55–58]) have been incorporated into AIDA online version 3 (AIDA online^3^).

#### 4.3.4. Other Possible Developments

There are other ideas for further work that warrant consideration. Eventually, if arrangements could be made to track users (teachers) at AIDA online, it would be possible to have a tutorial or series of lessons that teachers could go through to be credentialed to teach with the simulator. The idea could be developed to get teachers up to speed with using the diabetes simulations and what AIDA is about. Potentially, this could even form part of continued medical education (CME). Technically, it is likely to be less challenging to track users of an online simulator than a downloadable/offline simulator like AIDA v4.

One could even envisage a logon Perl script to monitor usage of AIDA online by patients as part of a RCT with HbA_1c_ measured before and after lessons with the simulator, possibly using home HbA_1c_ monitoring kits. Something similar (without HbA_1c_ monitoring) has been tried for a small number of medical students by DeWolf [47], but the approach might be more powerful with hundreds, or thousands, of people with diabetes accessing AIDA online and the accompanying Diabetes/Insulin Tutorial [46].

There have also been suggestions to permit some user selectable different colours to be chosen for the graphs in AIDA online. Allowing grey scale or other colour schemes to be selected may assist patients with visual impairments/colour blindness to maximize their access to and the usability of the web-based simulations, and this is planned to be investigated as part of future development work with AIDA online.

### 4.4. Conclusions

In terms of its functionality, the current version of AIDA online^2^ is similar in many ways to the PC version of AIDA (v4.3d). However, with its improved mouse-based user interface—and the fact that only one version of the program needs to be changed to distribute updates—it is envisaged that AIDA online^2^ may become more amenable to further upgrades and improvements in functionality, in due course.

It is recognised that making use of a simulator in a standard web-browser window, which most people know how to use, could also potentially help decrease the “learning curve” for generating simulations, compared with a downloadable version of the program.

Nevertheless, the designated purpose of the standalone PC software and AIDA online remains the same. In this respect, AIDA online should be solely regarded as an educational tool which some patients and HCPs have found fun and interesting to use. It is provided free of charge—from a not-for-profit website at: http://www.2aida.net—as a noncommercial contribution to continuing diabetes education, in the hope that more people may find it of some use.

A number of verification, validation, and clinical evaluation studies have been performed with the downloadable versions of AIDA v4 (e.g., see [15]). Thus far, relatively little evaluation work has been done with AIDA online, aside from a small study by DeWolf investigating the value of the web-based simulator as an educational tool for medical students [47] and an assessment of AIDA online for teaching high school students [64]. Part of the reason for the relatively limited assessment of the AIDA online version of the simulator, to date, may simply be its self-evident use.

However, in view of the fact that the mathematical model underlying AIDA online is the same as that in the downloadable software, going forward evaluation work for AIDA online may benefit from focusing particularly on human factor assessments, as well as identifying optimal ways to actually apply AIDA online in clinical/teaching settings.

One major advantage to utilising a server-based application (as opposed to a downloadable program) is the flexibility that is offered. New modules can be added to the simulator which become available instantaneously around the world. This has facilitated the development of AIDA online version 2 (AIDA online^2^), as well as various more recent refinements to the web-based simulator.

## 5. System Availability

The most up-to-date version of AIDA online (version 2) is available without charge at the http://www.2aida.net website. Following completion of further programming and bench testing work, it is expected that new, improved versions of AIDA online will become available in due course. People who wish to be automatically informed by email about updates to AIDA online are welcome to join the very low volume AIDA registration/announcement list by sending a blank email note to subscribe@2aida.org or by following AIDA on Facebook at http://www.facebook.com/www.2aida.org and/or at http://www.facebook.com/aida.diabetes.simulator1 or on Twitter at http://www.twitter.com/aida_diabetes.

## Figures and Tables

**Table 1:** Parameter pre-set values for the AIDA online (http://www.2aida.net) web-based diabetes simulator.

	Low/reduced	Normal	High/increased
RTG (mmol/L)	7	9	11
CCR (mL/min)	40	100	—
Hepatic insulin sensitivity	0.2	0.5	0.8
Peripheral insulin sensitivity	0.2	0.5	0.8

RTG: Renal threshold of glucose. CCR: Creatinine clearance rate.

**Table 2:** Gives typical values for file sizes (in kb) for data and image files made displayable/accessible by the AIDA online simulator. The small file sizes help explain the rapid data transfer via the internet, and contribute to the apparent speed of AIDA online. Reducing the file sizes with  .PNG files with the move to the AIDA online version 2 (AIDA online^2^) simulator in London, UK ensured reduced bandwidth and increased apparent speed for simulations.

	.JPG	.GIF	.PNG	.DAT
Preparation	8.7	1.6	1.2	
Plasma Insulin	15.1	2.8	1.5	2.3
Carbohydrate	9.2	1.7	1.3	
Glucose	15.1	2.3	1.3	2.2

JPG: Joint Photographics Experts Group; GIF: Graphics Interchange Format; PNG: Portable Network Graphics; DAT: ASCII text format data file.
